# CHINESE FAMILY CAREGIVERS’ EXPERIENCE SUPPORTING FAMILY MEMBERS WITH DEMENTIA TRANSITIONING INTO US NURSING HOMES

**DOI:** 10.1093/geroni/igae098.3934

**Published:** 2024-12-31

**Authors:** Yiyang Yuan, Catherine Dubé, Emily Lim, Shu Xu, Shan Qu, Emily McPhillips, Kate Lapane

**Affiliations:** University of Massachusetts Chan Medical School, Worcester, Massachusetts, United States; University of Massachusetts Chan Medical School, Worcester, Massachusetts, United States; University of Massachusetts Boston, Boston, Massachusetts, United States; University of Michigan, Ann Arbor, Michigan, United States; University of Massachusetts Boston, Boston, Massachusetts, United States; University of Massachusetts Chan Medical School, Worcester, Massachusetts, United States; University of Massachusetts Chan Medical School, Worcester, Massachusetts, United States

## Abstract

Asian American family caregivers of individuals with dementia experience a greater burden compared to other racial/ethnic groups, underscoring the need for formal long-term care options including nursing homes. Transitioning a family member with dementia into a nursing home can be stressful due to cultural expectations and the lack of linguistically and culturally appropriate care. This qualitative study focused on caregivers of Chinese descent, the largest Asian community in the U.S., and explored their experiences as they support their family members with dementia through the nursing home transition. We conducted fourteen in-depth, semi-structured interviews with Mandarin- or English-speaking family caregivers. Through thematic analysis, we identified six major themes: the role of filial piety, allocation of caregiving duties, reasons for and against nursing homes, decisions about nursing home care, the impact of the COVID-19 pandemic, and advice and suggestions. Filial piety remained a significant influence across all aspects of the caregiving experience. Caregivers reported physical, emotional, and financial struggles, limited availability of culturally and linguistically appropriate nursing home care, concerns about nursing home quality, and challenges in family discussions regarding the transition to a nursing home. Caregivers noted that COVID-19-related social isolation might have worsened their family members’ dementia. Many expressed the need for the Chinese community to become more familiar with healthcare options for the aging population and called for additional nursing home staff training to enhance the quality of culturally and linguistically appropriate nursing home care. Understanding caregivers’ experiences can also help identify areas for improvement in dementia care services.

